# Influence of Iodine Feeding on Microbiological and Physico-Chemical Characteristics and Biogenic Amines Content in a Raw Ewes’ Milk Cheese

**DOI:** 10.3390/foods7070108

**Published:** 2018-07-07

**Authors:** Maria Schirone, Rosanna Tofalo, Giorgia Perpetuini, Anna Chiara Manetta, Paola Di Gianvito, Fabrizia Tittarelli, Noemi Battistelli, Aldo Corsetti, Giovanna Suzzi, Giuseppe Martino

**Affiliations:** Faculty of Bioscience and Technology for Food, Agriculture and Environment, University of Teramo, Via R. Balzarini, 1, 64100 Teramo, Italy; rtofalo@unite.it (R.T.); giorgia.perpetuini@gmail.com (G.P.); acmanetta@unite.it (A.C.M.); digianvito.paola@gmail.com (P.D.G.); ftittarelli@unite.it (F.T.); noemi.battistelli@gmail.com (N.B.) acorsetti@unite.it (A.C.); gsuzzi@unite.it (G.S.)

**Keywords:** raw milk cheese, biogenic amines, iodine feed, physico-chemical composition

## Abstract

Iodine is an essential trace element involved in the regulation of thyroid metabolism and antioxidant status in humans and animals. The aim of this study was to evaluate the effect of ewes’ dietary iodine supplementation on biogenic amines content as well as microbiological and physico-chemical characteristics in a raw milk cheese at different ripening times (milk, curd, and 2, 7, 15, 30, 60, and 90 days). Two cheese-making trials were carried out using milk from ewes fed with unifeed (Cheese A) or with the same concentrate enriched with iodine (Cheese B). The results indicated that the counts of principal microbial groups and physico-chemical characteristics were quite similar in both cheeses at day 90. Cheese B was characterized by a higher content of biogenic amines and propionic acid. Propionic bacteria were found in both cheeses mainly in Trial B in agreement with the higher content of propionic acid detected.

## 1. Introduction

Milk and dairy products represent the second most important source of iodine in the European Union or in the United States [1] particularly for infants and children. Iodine deficit in the diet causes various thyroid dysfunctions and infant mortality [2]; iodine has a recommended daily intake of 150 µg for both adolescents and adults [3,4]. The concentration of this element in milk and dairy products has been reported in different papers and it can vary in terms of animal feed, the season (the higher concentration is in winter), and exposure to iodophors [2]. Changes in animal feeding have been proposed as one of the most promising approaches to modify iodine content in milk [5]. Some studies [6,7] have been carried out to evaluate the effects of dietary iodine supplementation in dairy goats and cows on milk iodine content and milk production traits. Nudda et al. [6] reported that the iodine supplementation in dairy goat diets doubled the milk iodine content when compared with the control group, even if no evident effect was observed in the gross composition of milk. On the contrary, Weiss et al. [7] found that iodine concentration increased in serum but not in milk after supplementation of this element in diets of dairy cows. In fact, very little information is available about the effects of iodine addition on ewes’ milk and milk-based product composition, nor about the response of dairy product microbiota.

Pecorino Incanestrato di Castel del Monte (ICM) is an artisanal semi-hard pasta filata cheese obtained starting from ewes’ raw milk without the addition of starter cultures. ICM is produced in the Abruzzo region (Central Italy) and is included in the list of typical products (PAT—Prodotti Agroalimentari Tradizionali). As other raw milk cheeses, the characteristics of the final product are influenced by several parameters such as raw milk microbiota, microorganisms deriving from equipment and from the dairy environments, and outside and inside grazing animal feeding systems [8].

In this study, the effect of dietary iodine supplementation in dairy ewes on biogenic amine (BA) content as well as microbiological and physico-chemical characteristics in ICM cheese was evaluated.

## 2. Materials and Methods

### 2.1. Cheese-Making Procedure

Cheese samples were manufactured in a small factory, located in the production area of ICM (L’Aquila, Abruzzo Region, Italy), from raw whole ewes’ milk of one or two daily milking without the addition of natural or commercial starter cultures. The milk was filtered and heated at 35–40 °C for 15–25 min and coagulated with lamb rennet at 38 °C, according to routine manufacture. Afterwards, the curd was broken and fit into special baskets, the so-called *fiscelle*. The product was salted and ripened up to 3 months. The final products weighted about 2 kg. Two different cheese-making trials were carried out in triplicate using milk (100 L). In a completely randomized block design, 2 groups of 15 Sopravissana ewes were assigned to 2 diets. In the first group, ewes were fed with unifeed (hay and concentrate) (Cheese A), while in the second group ewes were fed with unifeed enriched with iodine at a final concentration of 10 mg/kg (Cheese B). This concentration of iodine was selected according to Regulation EC No. 1459/2005 [9]. The cheese yield was about 24% in both cheese-making trials. Analyses were performed in triplicate on milk, curd, and cheese samples at different ripening times: 2, 7, 15, 30, 60, and 90 days.

### 2.2. Microbiological Analyses

Milk and cheese samples (10 mL or g) were diluted in 90 mL of a sodium citrate (2% *w*/*v*) solution and homogenized with a Stomacher Lab-Blender 400 (Steward Medical, London, UK) for 1 min. Serial dilutions in sterile peptone water (0.1% *w*/*v*) were plated in triplicate on different media to enumerate the following microorganisms: mesophilic lactobacilli, lactococci, aerobic mesophilic bacteria (AMB), yeasts, *Enterobacteriaceae,* enterococci, and coagulase-negative staphylococci (CNS), according to Schirone et al. [10]. The presence of *Escherichia coli* O157:H7, *Salmonella* spp., and *Listeria monocytogenes* was determined according to standard methods reported in ISO [11,12,13].

For the detection of propionibacteria, a semi-quantitative approach was applied as described previously [14,15]. DNA was extracted using PowerSoil DNA Isolation Kit (MoBio Laboratories) according to manufacturer’s protocol starting from 5 g of cheese as previously described [14]. PB1 (5′-AGTGGCGAAGGCGGTTCTCTGGA-3′) and PB2 (5′-TGGGGTCGAGTTGCAGACCCCAAT-3′) primer set was used. PCR amplification program consisted of denaturation at 94 °C for 4 min, 40 cycles of denaturation at 94 °C for 30 s, annealing at 70 °C for 15 s, and extension at 72 °C for 1 min followed by a final extension at 72 °C for 5 min.

### 2.3. Gross Physico-Chemical Composition

A radial slice of each cheese was randomly taken and used for physico-chemical assays. The rind of each slice was carefully removed, and the rind-less material was fully shredded. pH, water activity (a_w_), dry matter, total protein, fat, and ash content were determined according to Schirone et al. [9]. Iodine concentration was evaluated using a commercial kit according to manufacture instructions (Celltech, Turin, Italy) in milk and cheese samples.

Organic acids (mg/g) were determined as reported by Tofalo et al. [15] and Bouzas et al. [16] using an HPLC 200 series (Perkin Elmer, Monza, Italy) connected to a UV VIS detector at 210 nm. ROA Organic Acid H^+^ column (Phenomenex, Bologna, Italy) was used for the analyses. All determinations were performed isocratically with a flow rate of 0.7 mL/min at 65 °C using H_2_SO_4_ solution 0.009 N as mobile phase.

The nitrogen fractions determined were water-soluble nitrogen (WSN, expressed in %N) [17], trichloroacetic acid-soluble nitrogen (12% TCA-SN, expressed as %N) [18], and amino acid nitrogen (AAN, expressed as mg leucine/g) [19].

### 2.4. BA Determination

Determination of BA (mg/kg) was carried out as described by Schirone et al. [20]. In brief, 10 g of cheese samples were extracted and derivatized with dansyl chloride (Fluka Chimica, Milan, Italy). The chromatographic system consisted of an HPLC Waters Alliance (Waters SpA, Vimodrone, Italy), equipped with a Waters 2695 separation module connected to a Waters 2996 photodiode array detector. The separation of the analytes was carried out using a Waters Spherisorb C18 S3ODS-2 column (3 µm particle size, 150 mm × 4.6 mm Inner Diameter) equipped with a Waters Spherisorb S5ODS2 guard column. A linear gradient made up of acetonitrile and ultrapure water was applied: acetonitrile 57% (*v*/*v*) for 5 min; acetonitrile 80% (*v*/*v*) for 4 min, acetonitrile 90% (*v*/*v*) for 5 min. The peaks were detected at 254 nm.

### 2.5. Statistical Analyses

Statistical analyses were performed using the software STATISTICA for Windows (STAT. version 8.0, StatSoft Inc., Tulsa, OK, USA). Collected data were subjected to two-way analysis of variance (ANOVA) to detect significant differences. The principal component analysis (PCA) was performed on physico-chemical and microbiological data after auto-scaling.

## 3. Results

### 3.1. Microbial Analyses

Microbial counts are shown in Table 1. Overall, mesophilic lactobacilli, lattococci, AMB, enterococci, and yeasts showed a significant increase during the first days ripening. This was partly due to both microbial growth during coagulation and the physical retention of microorganisms in curds. The count of AMB obtained from the milk was higher in Cheese A (6.9 log CFU/mL) than in Cheese B (5.5 log CFU/mL) and then increased up to 8.4 log and 8.7 log CFU/g at 90 days of ripening, respectively. These counts are common in cheeses produced from raw milk, and they agree with those obtained in different cheese varieties such as Montasio [21] or Cebreiro [22,23].

Lactic acid bacteria (LAB) dominated in ICM cheeses during all ripening. In Cheese B, a higher number of lactococci and mesophilic lactobacilli was observed than in the Cheese A during the first stages of ripening, while at the end of ripening both cheeses showed similar counts, more than 8 log CFU/g. In general, LAB dominated the adventitious microbiota prevailing in all cheeses. Overall, in the early phase of manufacture, non-starter lactic acid bacteria (NSLAB) were present at very low values, whereas during ripening they increase from approximately 2.0 to 6.0 log CFU/g in ripened cheese [24]. Enterococci counts during ripening resulted to be quite different in ICM Cheeses A and B. In Trial A, their number increased from 2.8 log CFU/mL in milk up to a maximum value of 6.5 log CFU/g at 2 days and then decreased at 5.5 log CFU/g at the end of ripening. In Trial B, the counts started from 2.5 log CFU/g in milk, increased up to 6.3 log CFU/g after 15 days, and then decreased at 3.8 log CFU/g at 90 days of ripening. Enterococci represent the major part of curd microbiota and in some cases, they are the predominant microorganisms in the fully ripened product, constituting about the 41% of the LAB population [25]. In particular, enterococci have been recognized as an essential part of the natural microbial population of many dairy products, where they can sometimes prevail over lactobacilli and lactococci [22,26,27]. High levels of enterococci observed in other cheeses have been suggested to have a relevant role during the whole ripening process, because of their proteolytic and lipolytic activities that contribute to aroma compounds production (C4 metabolites such as diacetyl acetoin or 2,3-butanediol) [28,29].

As regards *Enterobacteriaceae*, they are associated to the natural microbiota of many dairy products, and together with coliforms are considered indicators of the microbiological quality of cheese. These microorganisms were present in milk of both cheeses and after 15 days of ripening, ranging from about 4.5 to 5.8 log CFU/g for Cheeses A and B respectively, whereas they were not enumerable (<10 CFU/g) in Cheese B at 90 days of ripening. *Enterobacteriaceae* are generally considered as microorganisms with a high decarboxylase activity, particularly in relation to the production of cadaverine and putrescine [30] and are common in many traditional cheeses of Mediterranean area [31]. The counts of CNS were higher in Milk B (6.3 log CFU/mL) than in Milk A (4.9 log CFU/mL). These microorganisms increased during the first days of ripening and decreased at 90 days with values of 3.4 and 4.3 log CFU/g in Cheeses A and B, respectively.

Yeasts, absent in milk in both trials, were present in curd and reached values of 4.4 and 5.1 log CFU/g in Cheeses A and B, respectively, at the end of ripening. Similar data have been reported in other raw milk cheeses such as Pecorino di Farindola [32,33].

Pathogens such as *Salmonella* spp., *L. monocytogenes*, and *E. coli* O157:H7 resulted absent in all the examined samples.

### 3.2. Gross Physico-Chemical Composition

The physico-chemical parameters and organic acids content for the two cheese-making procedures at 90 days of ripening are reported in Table 2. After 2 days of ripening, the pH values were 5.75 and 5.44 in Cheeses A and B, respectively (data not shown), and they then slightly decreased at the end of ripening. These differences, generally attributed to the metabolic activity of different species and strains of LAB, are typical of low acidified cheese produced with ewes’ raw milk [10]. The mean a_w_ values decreased as ripening progressed and at day 90 they were similar in both cheeses (about 0.97). A higher percentage of fat was observed in Cheese B (51.69% *w*/*w*) than in Cheese A (47.03% *w*/*w*) at the end of ripening, whereas proteins were present in the amount in Cheese A (44.12% *w*/*w*) than in Cheese B (40.92% *w*/*w*), even if no statistical differences were observed (*p* < 0.05). The average values of iodine concentration were 86.1 and 481.3 µg/100 mL in Milks A and B, respectively. At day 90, the iodine concentration was 128.7 μg/100 g in Cheeses A and 375.9 μg/100 g in Cheese B. The iodine amount in milk has been reported to reflect the dietary iodine content, and it is an indicator of the iodine status of the animal [34]. The iodine concentration in milk is directly proportional to the iodine levels in feedstuffs. Moreover, the season of milk production and fat content of milk can significantly affect its rate [35]. Manca et al. [36] found that iodine supplementation did not influence the goat milk fatty acid profile, except for some short-chain fatty acids. Milk fat and protein content did not vary between two groups of dairy sheep fed with iodine supplementation in diets at different concentrations [37].

Lactic acid was the most abundant organic acid with values of about 35 mg/g in both Cheeses A and B (Table 2). Similar concentrations of citric, acetic, and succinic acids were detected in both cheeses with values of about 0.5, 0.9, and 0.2 mg/g, respectively. Propionic acid was present in higher concentration in Cheese B. Therefore, to verify the origin of this organic acid, a genus-specific PCR was used. *Propionibacterium freudenreichii* was the only propionic bacteria detected, as demonstrated by the presence of a specific fragment of 850 bp. It was present in all samples, the only exception being Cheese A at 7 days of ripening (data not shown). Band intensities were correlated to propionic bacteria abundance. In Cheese A, the *P. freudenreichii* presence ranged from 10 to 10^3^ CFU/g—in Cheese B, from 10^3^ to 10^6^ CFU/g. Similar results were found by Tofalo et al. [15] in a traditional Abruzzo cheese where the presence of *P. freudenreichii* has been shown to play an important role in its sensorial characteristic and aromatic quality conferring an intense flavor.

**Figure 1:**
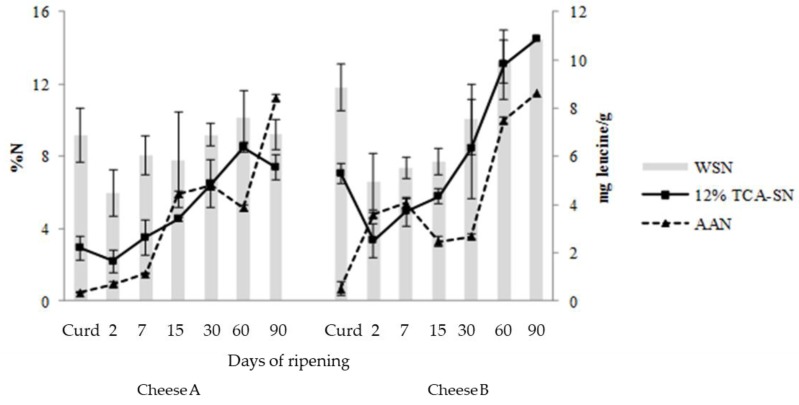
Evolution of nitrogen fractions during ripening in Cheeses A and B. WSN: water-soluble nitrogen; TCA-SN: trichloroacetic acid-soluble nitrogen; AAN: amino acid nitrogen.

Assessment of proteolysis in Cheeses A and B, through the determination of WSN, 12% TCA-SN and AAN over three months of ripening, is reported in Figure 1. The WSN value was 9%N in Curd A and about 12%N in Curd B. During the first weeks of ripening, there were no statistically significant differences between the examined cheeses in the level of WSN and the concentrations increased with a more intense rise in Cheese B, reaching a final rate of 14.5%N at day 90. The effect of feeding system on nitrogen fractions was more marked starting from 30 days of ripening, probably due to the impact of the milk as a source of microbial enzymes. The amount of 12% TCA-SN also increased progressively in both cheeses, but it was stronger always in Cheese B. Starter LAB and non-starter LAB (NSLAB) proteinases are principally responsible for the formation of 12% TCA-SN [38], that contains small peptides (2–20 residues) and free amino acids [39]. The average content of AAN showed a general similar evolution in both cheeses, but Trial A showed a slower proteolytic activity than that in Trial B. However, the final values obtained were similar: 8.42 and 8.60 mg leucine/g, respectively, for Cheeses A and B at 90 days of ripening.

### 3.3. BA Content

**Figure 2:**
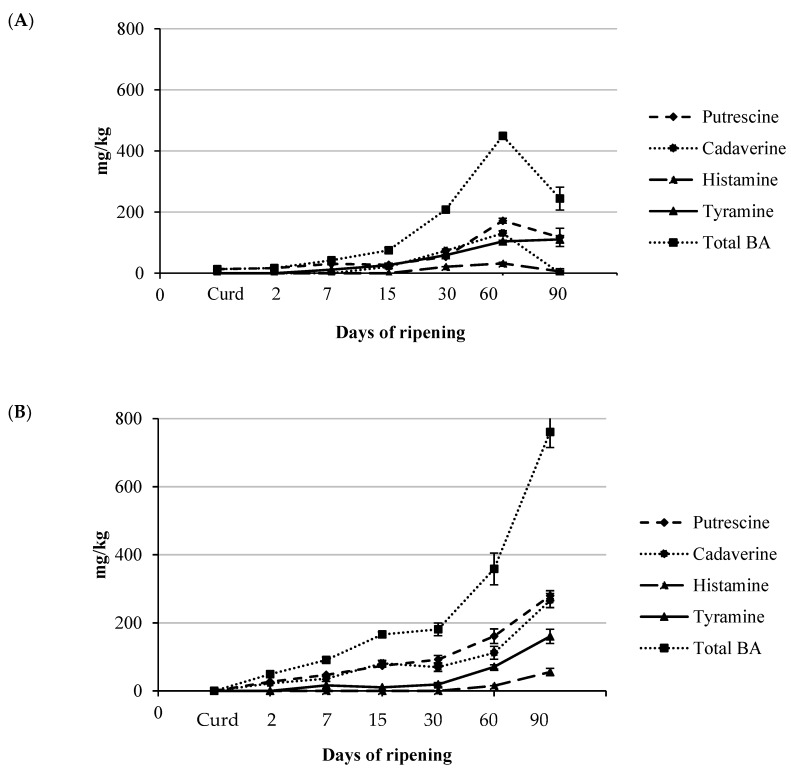
Biogenic amines content (mg/kg) during the ripening in Cheese A (**A**) and Cheese B (**B**). BA, biogenic amine.

The high content of BA in cheese is well documented [20,40]. The accumulation of BA has been mainly ascribed to the activity of NSLAB, even if an indirect role of LAB proteolytic activity could be hypothesized providing the precursor amino acids used for BA synthesis. Moreover, some factors, such as environmental hygienic conditions, decarboxylase microorganisms, and temperature and ripening of cheese can contribute to the qualitative and quantitative BA profiles [31]. The accumulation of BA at high concentrations and the presence of BA-producing microorganisms cannot be avoided in raw milk cheeses as well as in fermented foods and beverages. Total BA content was found to be similar up to 60 days in both cheeses with an average content of about 400 mg/kg (Figure 2A,B ). In raw milk A and B, only putrescine was detected at low concentrations (about 2 mg/kg). At day 60, the main amine was putrescine, followed by cadaverine, tyramine, and histamine. In Cheese B, a significant increase was observed in the total BA content at day 90 (760.7 mg/kg); in Cheese A, a decrease was detected at that time (244.30 mg/kg). The reduction was particularly evident for histamine (5.80 mg/kg) and cadaverine that disappeared. This fact could be explained by the presence of some BA-degrading strains, as reported by other authors [41,42,43]. Recently, Alvarez et al. [41] reported that a significant alternative to reduce BA content in fermented foods (such as cheese, wine, and sausages) was the use of BA-degrading strains, isolated from different origins. Fresno et al. [42] suggested that the addition of two strains (*Lactobacillus casei* 4a and 5b) were able to reduce BA contents in a Cabrales-like mini-cheese manufacturing model, although the exact mechanism via which this occurs remains unknown. In order to identify the pathways involved in the catabolism of these compounds, Ladero et al. [43] reported the draft genome of the *L. casei* 5b strain isolated from cheese. The use of BA-degrading strains could be particularly useful during cheeses manufacturing from raw milk in which a specific non-starter microbiota is essential for the organoleptic characteristic of the final product.

**Figure 3:**
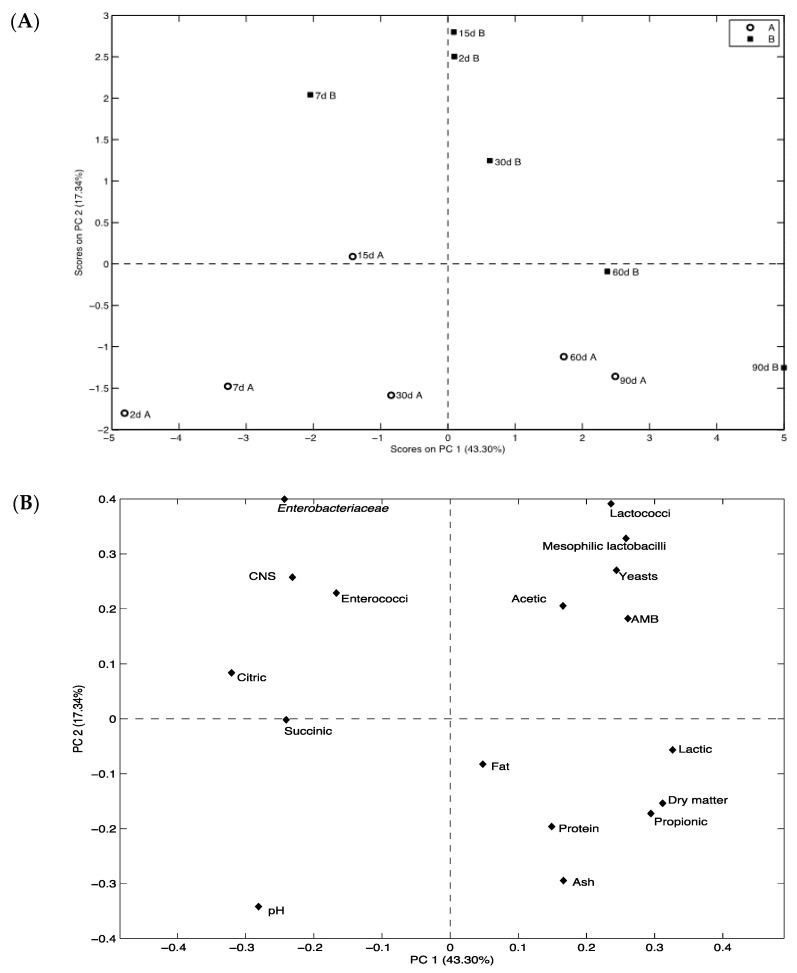
Score plot (**A**) and loading plot (**B**) of the first and second principal components (PCs) after PC analysis encompassing microbiological and physico-chemical parameters.

In order to understand the variability between the two different cheeses, PCA was carried out using as variables physico-chemical and microbiological data. The PCA results were shown in Figure 3, the score plot (A) and the loading plot (B). The two principal components (PCs) captured 60.64% of total variance in the first two dimensions with 43.30% and 17.34% explained by Factors 1 and 2, respectively. In the score plot (A), both Cheeses A and B at days 60 and 90 of ripening clustered together in the positive section of PC 1 and were closely related to the high values of dry matter, fat, protein, and ash content as well as propionic and lactic acids; meanwhile, the negative counterpart of PC 1 was mainly associated to the succinic acid and grouped the samples of Cheese A in the first month of ripening. The different ripening times of Cheese B were discriminated over the first PC, based above all on the counts of microorganisms.

## 4. Conclusions

The overall management system of the farm was the same, so it is possible to hypothesize that iodine influenced the main features of Pecorino Incanestrato di Castel del Monte cheese. The physico-chemical and microbiological data highlighted a relevant effect of dietary iodine supplementation on ewes’ raw milk and cheese microbiota. Even if the counts of principal microbial groups were quite similar in both cheeses, differences were found in some biochemical activities of microorganisms such as proteolytic/peptidasic activities or total BA content at day 90 of ripening. The findings call for a deep study on the selective effect of iodine on microbial populations in raw milk cheeses.

## Figures and Tables

**Table 1:** Evolution of principal microbial groups during the ripening in the two different trials expressed as log CFU/g.

Microbial Groups	Trial	Milk	Curd	2 Days	7 Days	15 Days	30 Days	60 Days	90 Days
Mesophilic lactobacilli									
	A	5.2 (0.02) *	6.6 (0.006) *	7.2 (0.01) *	7.8 (0.002) *	8.3 (0.02) *	7.2 (0.009) *	8.4 (0.006) *	8.7 (0.001) *
	B	6.8 (0.003) *	7.9 (0.001) *	8.6 (0.004) *	8.5 (0.004) *	8.8 (0.002) *	8.5 (0.002) *	8.1 (0.09) *	8.7 (0.001) *
Lactococci									
	A	5.4 (0.006) *	6.1 (0.013) *	7.5 (0.01) *	7.6 (0.01) *	8.1 (0.02) *	7.9 (0.004) *	8.7 (0.05) *	8.5 (0.004) *
	B	6.8 (0.001) *	8.4 (0.007) *	8.6 (0.005) *	8.7 (0.001) *	8.8 (0.001) *	8.8 (0.002) *	8.5 (0.05) *	8.5 (0.007) *
AMB ^a^									
	A	6.9 (0.001) *	6.7 (0.001) *	7.9 (0.001) *	7.4 (0.007) *	8.6 (0.004) *	8.9 (0.001) *	8.4 (0.001) *	8.4 (0.006) *
	B	5.5 (0.01) *	7.0 (0.014) *	8.7 (0.002) *	8.8 (0.004) *	8.9 (0.001) *	8.9 (0.001) *	8.9 (0.09) *	8.7 (0.006) *
Yeasts									
	A	-	4.3 (0.005) *	3.5 (0.007) *	4.6 (0.002) *	4.5 (0.006) *	4.4 (0.005) *	4.5 (0.004)	4.4 (0.007) *
	B	-	3.5 (0.003) *	4.7 (0.004) *	4.4 (0.004) *	5.2 (0.009) *	5.1 (0.009) *	5.3 (0.4)	5.1 (0.003) *
*Enterobacteriaceae*									
	A	3.4 (0.009) *	5.4 (0.007) *	4.7 (0.003) *	4.3 (0.009) *	4.5 (0.007) *	2.6 (0.005) *	2.4 (0.002) *	2.4 (0.004)
	B	3.4 (0.001) *	5.4 (0.005) *	5.8 (0.002) *	5.8 (0.001) *	5.8 (0.001) *	3.8 (0.002) *	3.9 (0.003) *	<1
Enterococci									
	A	2.8 (0.001) *	4.7 (0.004) *	6.5 (0.004) *	5.3 (0.002) *	4.5 (0.004) *	4.3 (0.004) *	5.8 (0.003) *	5.5 (0.003) *
	B	2.5 (0.006) *	5.7 (0.002) *	5.6 (0.005) *	6.0 (0.001) *	6.3 (0.002) *	6.1 (0.001) *	5.1 (0.07) *	3.8 (0.001) *
CNS ^b^									
	A	4.9 (0.002) *	6.4 (0.003) *	5.4 (0.003) *	6.5 (0.001) *	6.5 (0.003) *	6.7 (0.001) *	3.1 (0.003) *	3.4 (0.01) *
	B	6.3 (0.005) *	6.4 (0.003) *	7.3 (0.007) *	6.8 (0.001) *	5.7 (0.002) *	5.6 (0.003) *	5 (1) *	4.3 (0.01) *

The data are reported as mean (S.D.); samples for each microbial group at the same ripening time marked with * showed statistically significant differences (*p* < 0.05). ^a^ aerobic mesophilic bacteria, ^b^ coagulase-negative staphylococci.

**Table 2:** Physico-chemical characteristics and organic acids content (mg/g) in cheeses at the end of ripening.

Parameters	Cheese A	Cheese B
**Physico-chemical**		
pH	5.55 ± 0.20	5.39 ± 0.10
a_w_	0.98 ± 0.01	0.97 ± 0.01
% Dry matter	67.68 ± 4.80	66.15 ± 2.23
% Fat ^1^	47.03 ± 3.13	51.69 ± 1.73
% Protein ^1^	44.12 ± 6.68	40.92 ± 7.54
% Ash ^1^	8.85 ± 0.26	7.39 ± 0.47
**Organic acids**		
Citric acid	0.5 ± 0.1	0.40 ± 0.07
Succinic acid	0.10 ± 0.05	0.21 ± 0.03
Lactic acid	35 ± 4	36 ± 4
Acetic acid	0.80 ± 0.05	0.90 ± 0.06
Propionic acid	0.04 ± 0.03	0.13 ± 0.06

Data are expressed as mean ± S.D.; ^1^ these parameters (fat, protein, and ash) are expressed in dry matter; no statistically significant differences were observed (*p* < 0.05).
